# cds46, a highly variable carp edema virus gene

**DOI:** 10.1099/jgv.0.002048

**Published:** 2024-11-20

**Authors:** Laetitia Montacq, Doriana Flores, Hélène Giummarra, Laurane Pallandre, Anaïs Angot, Rodolphe Thomas, Amélie Charrier, Laurie Lamothe, Mélanie Lesne, Carine Bellet, Nicolas Keck, Françoise Pozet, Aurélien Tocqueville, Sophie Le Bouquin-Leneveu, Jésabel Laithier, Jean K. Millet, Stéphane Bertagnoli, Marine Baud, Laurent Bigarré

**Affiliations:** 1 IHAP, Université de Toulouse, INRAE, ENVT, 31300 Toulouse, France; 2 Laboratoire de Ploufragan-Plouzané-Niort, ANSES, 29280 Plouzané, France; 3 Laboratoire de Ploufragan-Plouzané-Niort, ANSES, 22440 Ploufragan, France; 4 Laboratoires des Pyrénées et des Landes, 40004 Mont-de-Marsan, France; 5 Laboratoire Départemental Vétérinaire, CS 69013, 34967 Montpellier, France; 6 Laboratoire Départemental d’Analyses LDA39, 59 rue du Vieil Hôpital, 39802 Poligny, France; 7 ITAVI, Service Aquaculture, 76000 Rouen, France; 8 Université Paris-Saclay, INRAE, UVSQ, Virologie et Immunologie Moléculaires, 78352 Jouy-en-Josas, France

**Keywords:** sleepy disease, carp, *Poxviridae*, epidemiology

## Abstract

Carp edema virus disease (CEVD) is a severe viral illness that causes substantial economic losses in wild and farmed common carp and koi. It is caused by carp edema virus (CEV), a member of the *Poxviridae* family*,* whose genetic diversity and genome evolution are poorly understood. Based on a genomic fragment of the *4a* gene, two genogroups, genogroup I (gI) and genogroup II (gII), have been identified in samples of different origins. By analysing a series of recent samples, we highlight here a new genomic region of interest that varies by substitutions, indels and putative recombinations. In the Japanese reference sequence, this region encodes an ORF, cds46, whose function is unknown despite weak homologies with genes of some members of the *Iridoviridae*. Surprisingly, AlphaFold protein structure prediction analyses link cds46-encoded ORF with cellular endonucleases, providing insights into its possible origin. The ORF is absent in all gI haplotypes and in some gII haplotypes. Apart from the absence of cds46, gI haplotypes show an insertion of 121 bp with no homology to any viral sequence. When present, cds46 showed two groups of alleles differentiated by substitutions. The analysis of the cds46 locus showed that some samples from fish batches contained mixes of different haplotypes, irrespective of their origin (i.e. France, Japan or Israel). In a 2023 sample, we also found a virus carrying a gII-like atypical *4a* allele first identified in France in 2015, indicating the limited but persistent spread of this virus in the country. The cds46 locus is a new target that may be useful for identifying and tracking CEV haplotypes.

## Data availability

The authors confirm that all supporting data, including GenBank accession numbers and protocols, have been provided within the article or through supplementary data files.

## Introduction

For nearly four decades, carp edema virus disease (CEVD) has been steadily emerging worldwide as a major disease, affecting *Cyprinus carpio* and its various varieties, mainly koi and common carp (CC) [1,3]. Also known as koi sleepy disease (KSD), this illness has resulted in massive mortality rates – up to 100% in farmed koi and 80% in common and mirror carps in ponds [4,5]. One typical clinical sign is lethargy, with fish often resting on the tank or pond bottom, or on the surface, depending on various factors including growth stage [6,7]. Other clinical signs may include excessive mucus on the skin and the gills, as well as enophthalmia. Water temperature is an important parameter in the expression of the disease, with outbreaks occurring at different temperatures depending on undetermined factors [8]. For instance, in Japan, KSD typically occurs between 15 and 25 °C. In Europe, outbreaks are most common post-winter, with CC being most affected when the water temperature rises, typically from 6 to 15 °C. Nevertheless, outbreaks have also been reported in late summer when water temperatures reach 22 °C [9].

The disease is caused by carp edema virus (CEV), a member of the *Poxviridae* family [10]. Due to extensive fish trade, CEV circulates widely within and between regions, including internationally. Fish often carry the virus without showing clinical signs, and the disease can be triggered by stressors, such as moving from one pond to another, or changes in water temperature. When an outbreak occurs, it is important to trace the origin of the virus to prevent future introductions [11]. Therefore, viral genetic markers are essential for linking a virus to those characterized in previous studies.

A major breakthrough in the genetics of CEV occurred in 2021 with the complete genome sequence of a virus from koi originating from Japan [12]. This study revealed a genome consisting of a large dsDNA molecule, almost 460 kbp in size, confirming an earlier estimation obtained by pulsed-field electrophoresis [13]. This genome encodes a large number of open reading frames (ORFs), some with putative functions inferred from homologous genes found in other large DNA viruses, but most with unknown functions. Despite this key study, knowledge of the genes necessary for molecular characterization remains in its infancy.

Nevertheless, at least two genogroups, genogroup I (gI) and genogroup II (gII), have been identified based on a small portion of a single gene, the *4a* gene, often called *p4a* since it encodes a precursor of the 4A protein [14]. Generally, viruses found on CC or mirror carps are grouped into gI, and those found in koi belong to gII. Based on a larger fragment of *4a*, the gI/gII demarcation has been confirmed and refined by the finding of two molecular markers in addition to substitutions [3]. One marker, M1, contains a few single-nucleotide polymorphisms (SNPs) and a very short insertion/deletion (indel) located in a microsatellite. M1 has three alleles, two of which are specific to gII and one to gI. A second marker, M2, is represented by three alleles varying only by SNPs: one specific to each genogroup and a third found in an atypical haplotype. As a notable exception, this atypical viral haplotype from a CC collected in France in 2015 was highly related to a gII haplotype over a 747 bp stretch of *4a* but showed a significant number of substitutions compared with all the other CEV haplotypes over a shorter 143 bp stretch [3]. This finding suggests that this short segment originated from a virus of another, still unknown, genogroup that had recombined with a gII haplotype. More recently, in an effort to find other genetic markers of interest, two more genes were sequenced from Ukrainian samples [15]. However, their variability only confirmed the distinction between the two main genogroups.

Here, a new genomic region of interest, cds46, was serendipitously identified. It encodes a product of unknown function and exhibits genetic variations, including complete absence in some haplotypes compared to the reference genome, as well as substitutions and possibly also recombinations. The analyses based on AlphaFold prediction of cds46-encoded protein reveal an ancestral link with cellular endonucleases, which points to intriguing hypotheses on its origin and evolution.

## Methods

### Sampling

All fish samples (mostly gills) were collected in France from 2018 to 2023 during outbreaks of CEVD affecting koi or CC. The samples originated from diverse geographical locations and were collected over various years (Table 1). Samples 23-051 and 20-D1 were taken from the water in which 60 and 150 koi were imported from Japan in 2019 and 2020, respectively (travelling for 3 days), to a French farm. Two additional water samples (23-052 and 23-053) were taken from plastic bags containing 430 koi each, imported from the same Israeli farm and immediately sampled upon arrival at a second French farm in 2023. Sample 18-230B was a pool of koi fish tissue held in a plastic bag sent by a French farmer to the ANSES laboratory in 2018. This sample belongs to the same batch as the pool of fish 18-230A characterized in a previous study [3]. Four samples of CC were obtained from four ponds in the North region of France that experienced massive CC mortalities (up to 1 t) in spring 2023 (Table 1). The ponds were each distant of several kilometres one to another, without hydric connection. They were all restocked with fish several weeks before the mortalities: two with CC and two with other fish species.

**Table 1.** Features of the samples analysed in this study. Note that sample 18-230B has the same origin but is distinct of sample 18-230b characterized in a previous paper. nd, not determined

Sample code	Year of sample	Sample	Carp variety	PCR signal (Ct)	Country of origin	Genotype *4a*/cds46	First sample description
18-230B	2018	Tissues (gills)	Koi	26.1	France	gII/A1+A2+BΔ1	This article
19-115	2019	Tissues (gills)	Common	23.8	France	gI/CΔ1	[3]
20-237	2020	Tissues (gills)	Koi	22	France	gII/BΔ^nd^	[3]
21-226	2021	Tissues (gills)	Common	32.6	France	gI/CΔ1	This article
21-227	2021	Tissues (gills)	Koi	29.1	France	gII/BΔ^nd^	This article
21-228	2021	Tissues (gills)	Koi	31.6	France	gII/A1+BΔ^nd^	This article
22-142	2022	Tissues (gills)	Koi	24.4	France	gII/A1	This article
23-051	2019	Shipping water	Koi	28.3	Japan	gII/A1+BΔ1	This article
23-052	2023	Shipping water	Koi	29.5	Israel	gII/BΔ1	This article
23-053	2023	Shipping water	Koi	30.4	Israel	gII/A2+BΔ1	This article
23-066	2023	Tissues (gills)	Common	30.4	France	gI/CΔ1+CΔ2	This article
23-068	2023	Tissues (gills)	Common	24.8	France	gI/CΔ1+CΔ2	This article
23-069	2023	Tissues (gills)	Common	33.7	France	Atypical/BΔ2	This article
23-072	2023	Tissues (gills)	Common	22.2	France	gI/CΔ1	This article
20-D1	2020	Shipping water	Koi	22.3	Japan	gII/BΔ2	This article

### Nucleic acid extractions

For fish tissues, the total nucleic acids were extracted as previously reported using NucleoSpin® Virus (Macherey-Nagel) or ADIAMAG (BioX Diagnostics) kits [3]. For extracting nucleic acids from the water in shipping bags, two distinct methods were used. In 2019 (sample 23-051), 200 µl of water was extracted using One-4-All genomic DNA minipreps (Bio Basic Inc., Canada) according to the manufacturer’s instructions. In 2020 and 2022 (samples 23-052 and 23-053), a similar method was used, but with modifications to the lysis and membrane adsorption steps to increase the extracted volume to 1.5 ml. Volumes of 15 µl of Tris-HCl 1M, 79 µl of EDTA 0.5M and 75 µl of SDS 10% were added to 1.5 ml of thawed shipping water together with 200 µg of proteinase K (Macherey-Nagel). After an incubation step of 30 min at 55 °C, an equal amount (1.5 ml) of absolute ethanol was added. NucleoSpin® RNA Virus Mini Kit (Macherey-Nagel) columns were loaded with successive volumes of 650 µl until the entire sample was processed (the flow-through was removed after centrifugation). The subsequent steps of washing and elution were undertaken following the manufacturer’s instructions. The nucleic acids were finally eluted using 50 µl of the provided buffer.

### PCR targets

Samples were tested for CEV using a real-time PCR assay modified from Matras *et al.* [14] targeting the *4a* ORF. Primers CEVqFor1/CEVqRev1 were used at 500 nM in a final reaction of 25 µl containing 200 nM of CEV qProbe1 and 6.25 µl of the Fast Virus real-time 1-Step Master Mix (Applied Biosystems™). The cycles consisted of 20 s at 95 °C followed by 40 cycles of 15 s at 95 °C and 30 s at 60 °C. Samples were declared positive for Ct values below the detection threshold (37.6±0.5).

For CEV-positive samples, a region of the *4a* ORF was amplified using a newly designed set of primers (oPVP824/oPVP857) well adapted to all haplotypes of our collection, including those that proved recalcitrant to amplification using the CEVforB/oPVP322 pair of primers described elsewhere [3]. The new amplified portion varied between 1,074 and 1,077 bp and encompasses the portion of 890–893 bp studied with the previous set (sequences of primers in Fig. S1, available in the online version of this article). Each primer was used at 400 nM in the final PCR reaction, which included Platinum™ Taq DNA Polymerase High Fidelity (Invitrogen) and co-reactants, as previously described in ref [3]. The PCR cycles consisted of one denaturation step at 94 °C for 2 min, followed by 40 cycles at 94 °C for 15 s, 58 °C for 30 s and 68 °C for 1 min.

The complete cds46 was amplified using one of the three oligonucleotide pairs successively tested and proven to exhibit different levels of specificity (Fig. S1). The two sets, ogp46f/ogp46r and oPVP934/oPVP935, were functional on CEV from koi samples, but not – or were less efficient – on CEV from CC. Finally, a third newly designed set (oPVP944/oPVP946) was successfully used on all CEV-positive samples. Each primer was used at 400 nM in the final PCR reaction, which included one unit of Platinum™ Taq DNA Polymerase High Fidelity (Invitrogen) and co-reactants, as previously described [3]. The PCR cycles consisted of a denaturation step at 94 °C for 2 min, followed by 40 cycles at 94 °C for 15 s, 56 °C for 30 s and 68 °C for 1 min.

For all conventional PCRs (cPCRs), a volume of 10 µl of the reaction products was loaded on a precast 2% agarose E-Gel® (Invitrogen) for visualization. Due to sourcing issues, some of the gels were pre-stained with SYBR® Safe and others with SYBR® Gold, the latter being more sensitive, but associated with artefacts of DNA migration.

### Sanger and Oxford Nanopore Technologies sequencing

The PCR products were either purified by simple methods or TA-cloned before sequencing using Sanger-derived methods. Sequencing was carried out at the ANSES laboratory, as reported previously [3], or by the commercial GATC sequencing service (Eurofins). In some cases, several amplicon clones from a single sample were sequenced.

One amplicon from sample 18-230B was sequenced using a long-read method [Oxford Nanopore Technologies (ONT)] as previously described [16]. Briefly, 100 ng of purified amplicon was used to create a library with the ligation sequencing kit DNA V14 (ONT SQK-LSK114), according to the manufacturer’s recommendations. The library was read using a flongle (ONT) run for 4.5 h on a MinION platform. The sequence was decrypted using fast base calling.

### Phylogenetic analyses

The DNA sequences were aligned, and the levels of identities were calculated with Geneious Prime 2023 (Biomatters Ltd). For phylogenetic analysis, alignments were performed with the Clustal method implemented in mega7 [17]. Phylogenetic trees were generated using the maximum-likelihood method with 1,000 bootstrap replicates. For the *4a*-based trees, the sequences were oriented in the sense direction of the coding sequence (cds). For the cds46-based trees, the complete reference genome (GenBank accession number LC613089) was included alongside our sequences.

### Prediction of CEV cds46 structure and structural homology search

The amino acid (aa) sequence of CEV cds46 A1 allele sample 23-053 was used to generate a structural model of the protein using AlphaFold 3 (https://alphafoldserver.com) [18,19]. The model was colour coded using the predicted local distance difference test (pLDDT) confidence scoring metrichttps://pymol.org. The AlphaFold model of CEV cds46 protein was then used as a query in FoldSeek (https://search.foldseek.com) to search for structural homologues through several structural databases including the AlphaFold Database (AFDB) [20]. For Atlantic herring (*Clupea harengus*) hit (UniProt A0A6P8G392) and zebrafish (*Danio rerio*) hit (UniProt A5PM73), the Gene Ontology (GO) functional annotations as DNA/RNA non-specific endonucleases were found on UniProt (https://www.uniprot.org/). For zebrafish (*D. rerio*) hit (UniProt A8KB42), the gene annotation was inferred by performing a basic local alignment search tool (blast) search of the protein sequence (https://blast.ncbi.nlm.nih.gov). The closest match was a protein from zebrafish (NCBI NP_001121868) annotated as a DNA/RNA non-specific endonuclease. Molecular graphics, structural alignment analyses and visualizations were performed using UCSF ChimeraX [21].

## Results

### Variation of the cds46 region

From collections of CEV-positive samples stored in various French laboratories, 15 samples were selected for their diverse origins and natures. The samples included water samples from three batches of koi imported from Israel or Japan (Table 1). In particular, CEV was detected (Ct 22.2 to 33.7) for the first time in the North region of France, affecting four ponds where massive mortalities of CC (up to 1 t) occurred in 2023.

In our initial attempt to obtain genomic data of epidemiological interest from CEV, a set of nanopore-based long reads was produced, starting from the total DNA extracted from the shipping water used for the transport of infected koi from Japan to France (sample 20-D1). Despite the relatively high viral DNA load in the water (Ct 22.3), the number of CEV-related reads was insufficient to obtain a full genome coverage and significant depth (not shown). Nevertheless, three reads matching cds46 of CEV (LC613089) were identified. Surprisingly, these reads exhibited a large deletion, including the entire ORF, compared with the reference genome. To exclude the possibility of a sequencing artefact, this region was amplified using an initial specific set of primers (ogp46F/ogp46R, Fig. S1) at the National Veterinary School of Toulouse Laboratory and directly sequenced using the Sanger method. The absence of a 593 bp portion encompassing the complete cds46 ORF was confirmed by this new sequence. To determine if this deletion was exceptional or common in CEV genomes, samples of different origins were tested at the ANSES laboratory. Conventional PCR with the same set of primers was performed on six French samples from koi. The six samples yielded amplicons, yet three distinct patterns were observed following electrophoresis (data not shown). One pattern consisted of a single amplicon of about 1.2 kb (e.g. sample 22-142B) – a size expected from the intact reference sequence. The second pattern consisted of an amplicon of about 0.6 kb (e.g. sample 20-237). The third pattern was a mixture of the two types of amplicon (e.g. sample 18-230B). Sequencing of the amplicons confirmed their relationship to the CEV cds46 and revealed their exact sizes: 582 and 1175 bp. The 582 bp product corresponded to the deleted genomic region of 593 bp previously detected in sample 20-D1 (data not shown).

**Fig. 1.**
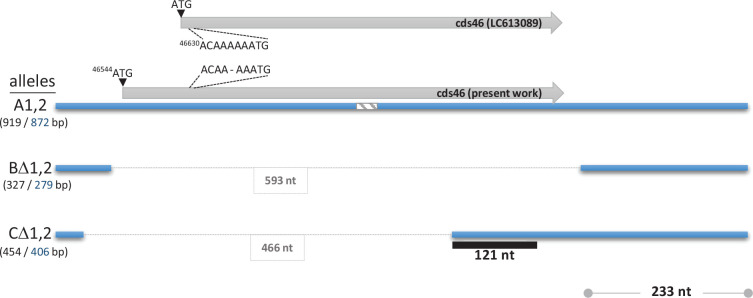
Genetic map of the cds46 region. The three sizes of amplicons produced with primers oPVP944/oPVP946 are indicated by the blue bars within the parentheses and the full-length sizes of the amplicons (including primers) (in black); the sizes of the sequence segments used to construct phylogenetic trees are shown in blue. A specific 233 bp region, common to all alleles, was used for a phylogenetic analysis (see text). The black bar indicates the segments of CΔ alleles with no homology to reference sequence of CEV. The striped bar is a 30 nt-long sequence used for selecting ONT read matching A alleles only (see text). The size (nt) of the deleted segment in alleles BΔ and CΔ is boxed.

Unfortunately, the initial set of PCR oligonucleotides, derived from a gII sequence, was ineffective in initiating DNA amplification across all CC samples, potentially due to the presence of multiple mismatches. Subsequently, a second set of primers (oPVP934/oPVP935) was tested (Fig. S1) and proved efficient to amplify the cds46 region from one CC sample (i.e. sample 19-115-3). Despite this success, the primers were nonetheless inefficient on other CC samples, again suggesting mismatches between the primers and their targets (not shown). The amplicon obtained from sample 19-115-3, with an apparent size of about 450 bp, was nevertheless sequenced. This sequence revealed a deletion of 466 bp compared with the reference cds46 and was slightly shorter than the previously mentioned complete cds deletion. Surprisingly, a domain of nearly 121 bp showed no apparent homology to any sequence, neither in the rest of the CEV genome nor to any in GenBank, as tested with blast (https://blast.ncbi.nlm.nih.gov/Blast.cgi) (Fig. 1).

**Fig. 2.**
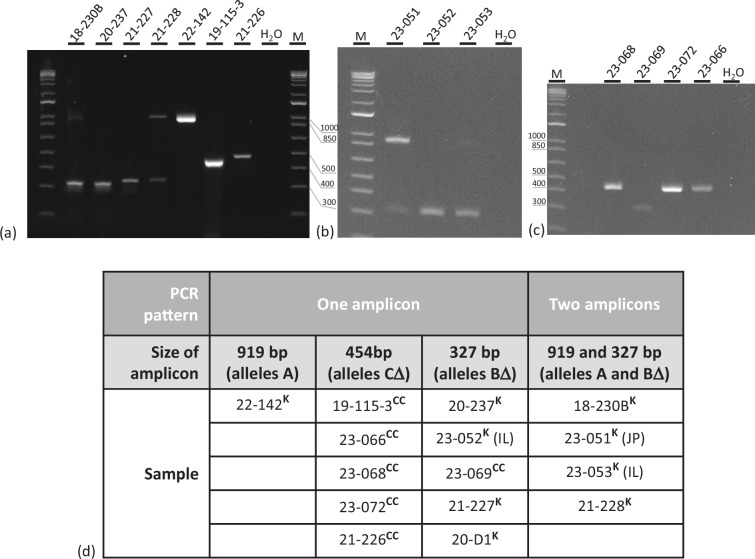
Amplification products of the cds46 region of carp samples of various origin. (a) Samples extracted from fish tissues or transport water. (b) Samples from transport water from Japan (JP, 23-051) or Israel (Il, 23-052 and 23-053). Sample 20-D1 is not shown. (c) Samples from the North region (France). (d) Overview of the number and sizes of the amplicons obtained in (a), (b) and (c), as well as sample 20-D1. M, 1 kb molecular ladder (Invitrogen); H_2_O, negative control of PCR; CC, common carp; K, koi. The left E-Gel™ is stained with SYBR™ Gold II and the two others with SYBR™ Safe (Thermo Fisher).

Based on this successful sequencing of a genomic fragment of virus 19-115-3 (from CC), we designed a new primer pair (oPVP944/oPVP946, Fig. S1) better adapted to viruses of both gI and gII and matching the cds46 region of all possible haplotypes. Using these primers, PCR assays readily produced amplicons for all 15 tested samples, regardless of their geographical origin (i.e. France, Japan and Israel) or host (CC or koi) (Fig. 2). Four distinct patterns were observed among the samples, consistent with the results mentioned earlier. Three patterns consisted of single amplicons of various sizes (327, 454 or 919 bp) (Figs1  2). These amplicons, located closer to the extremities of the cds46 ORF, were shorter than those obtained with the initial sets of primers. Upon sequencing, they were identified as alleles, exhibiting both SNPs and indels as described earlier. Allele types were tentatively named A for the 919 bp amplicon, BΔ for the 327 bp amplicon and CΔ for the 454 bp amplicon, with Δ indicating the cds46 deletion. The fourth pattern consisted of a mixture of alleles A and BΔ (919 and 327 bp).

Interestingly, the 454 bp amplicon (allele CΔ) was found exclusively in CC, and not in koi fish. Conversely, the 919 bp (allele A) and the co-occurring A and BΔ alleles (919 and 327 bp) were observed only in koi samples. The 327 bp (BΔ) allele alone was identified in four koi samples and also in sample 23-069 (CC) (Fig. 2). Therefore, based on this limited sampling, the different alleles of cds46 were strongly associated with different carp varieties.

**Fig. 3.**
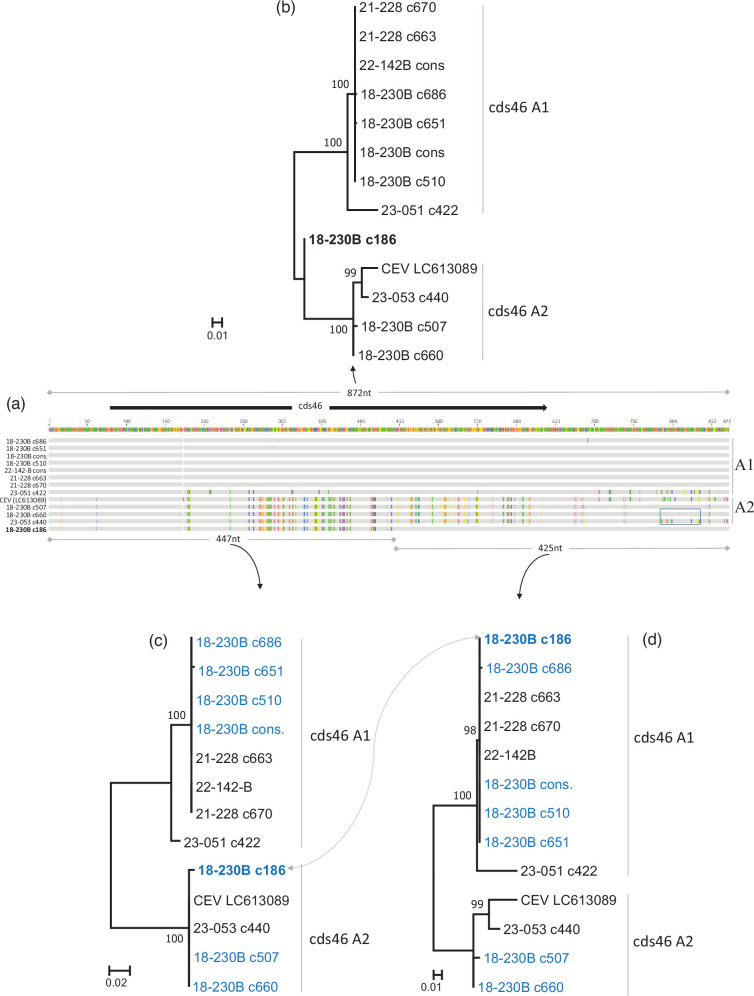
Alignment and phylogenetic analysis of cds46 A alleles. (a) Alignment of all the sequences of A alleles. (b) The phylogenetic tree above the alignment was constructed from the complete sequences. (c, d) Trees created using two sequence segments (left, 447 nt and right, 425 nt). All trees were obtained using the maximum-likelihood method (1000 replicates); bootstrap values less than 80% are not shown. Sequence names in blue are sequences from the same sample; in bold, the sequence exhibiting a hybrid pattern. Cons. indicates a consensus of several sequences. The boxed are of the alignment focuses on a region differing between two clones nearly identical in the rest of the sequences. All the samples of this figure are of the gII group according to the *4a* gene.

A phylogenetic analysis, based on the longest amplicon sequence (872 bp without primers), revealed two groups of alleles tentatively named A1 and A2, differing by SNPs and exhibiting identities varying between 86 and 100% (Fig. 3a, b). In particular, sample 18-230B showed variability among its seven individual cloned genomic fragments. Specifically, four clones clustered with A1 and two with A2 (Fig. 3b). The seventh sequence (c186) of sample 18-230B displayed a unique phylogenetic position, sharing over 99.3% identity with A2 sequences over the first 447 nt and 100% identity with A1 sequences in the remaining portion (Fig 3c, d). Therefore, depending on the sequence segment analysed, this clone’s position in the phylogenetic trees varied radically. This heterogeneity strongly suggests a hybrid haplotype between viruses of the two genogroups, possibly resulting from a recombination event. Upon visual inspection of the alignment of allele A sequences, two other clones exhibited chimeric patterns, indicating that they may also result from recombination events involving other viruses. For instance, sample 23-051 (c422) showed high homology with the reference genome over its 5′ and 3′ ends but differed markedly in its central portion of about 420 bp (Fig. 3a). Additionally, a 51 bp segment downstream of cds46 differed between clones 18-230B c660 and 23–053 c440, while the remaining sequences were highly conserved.

The presence of a single chimeric molecule (c186) among the longest cloned products raises the question of its real occurrence in the sample 18-230B. To estimate its frequency, a set of long-read sequences (ONT) was generated starting from a new PCR product obtained from sample 18-230B. Of the 153,454 reads obtained, a total of 137,317 reads aligned with the complete sequence of clone c186. As expected, these reads matched both the deleted (BΔ) and complete (A) alleles. Subsequent screening using a 30 nt-long motif specific to c186 (Fig. 1) identified only 18 reads, all showing perfect or nearly perfect identity (with at most, only 1 substitution). Apart from this motif, each of these 18 reads exhibited 10 to 73 SNPs or indels. These variations can possibly be attributed to errors during the sequencing process or to the quasi-species nature of the virus amplified from the mix of fish. Nonetheless, a consensus sequence of these 18 reads exhibited only 6 substitutions compared with c186. Two of these six changes were located in a poly-A motif prone to errors, and another was a non-determined base (A or T) consistent with the nt found in c186. In summary, the hybrid pattern of c186 initially detected using Sanger sequencing was further validated by Next Generation Sequencing across a new amplicon population, albeit at a very low frequency (0.01%). This sparse representation contrasts with the detection of one recombinant among the seven clones analysed with Sanger sequencing.

**Fig. 4.**
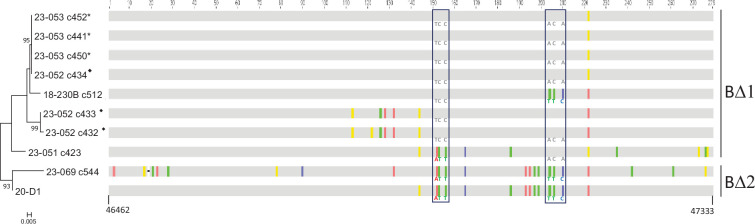
Alignment and phylogeny of the cds46 BΔ allele. Below the alignment, the first and last nts are numbered according to their position in the complementary sequence of the reference genome (GenBank LC613089). The position of each nt within the amplicon is shown above the alignment. The sequences with the same symbol (♦ or *) are from the same sample. The boxes highlight two triplets of nts, each with two alleles, linked to or dissociated from the other triplet allele according to the sample. For the maximum-likelihood analysis, bootstrap values less than 80% are not shown. Except sample 23-069, which is atypical, all the samples belong to gII according to the *4a* gene.

Six selected samples exhibiting cds46 BΔ alleles (279 bp without the PCR primers) were compared among each other (Fig. 4). The identity levels between the nt sequences (obtained either from direct amplicon sequencing or after amplicon cloning) varied between 90.3 and 100%. The most distinct sample was 23-069 (CC), differing by 20 to 27 nt from the other sequences. Interestingly, the sequence of sample 23-069 showed the highest similarity to 20-D1, with a match of 95%. Among individual PCR clones, three sequences from an Israeli sample (i.e. 23-053) were strictly identical. Additionally, three sequences from another Israeli sample (i.e. 23-052) exhibited some heterogeneity: two clones were nearly identical (1 SNP), and the third clone differed from the others by 6 or 7 nt. Remarkably, this latter clone exactly matched the three cloned sequences of sample 23-053. This suggests that sample 23-052 contained at least two slightly distinct haplotypes, one being common to 23-053. Upon scrutinizing the alignment of all BΔ sequences, several very short portions displayed patterns that might result from recombination events. For instance, two nt triplets each had two possible alleles, for which we observed four combinations, suggesting an occurrence of recombination (Fig. 4). In a phylogenetic analysis, two clusters were distinguished: the first, tentatively designated BΔ2, included 23-069 and 20-D1; the second, BΔ1, consisted of the remaining samples (Fig. 4). Nevertheless, the suspicion of recombination between the sequences weakens the reliability of this analysis.

**Fig. 5.**
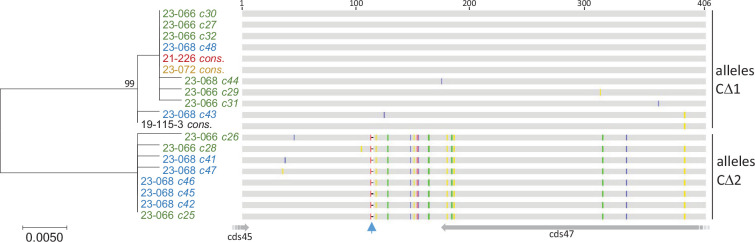
Alignments and phylogeny of sequences of cds46 CΔ alleles from CCs. Five samples (one colour per sample) exhibiting the 454 bp amplicon were analysed. The names of the five samples are followed either by the identification number of individual clones obtained from an amplicon or by the term *cons.* (consensus of nearly identical eight to ten clones). The position of the nts within the amplicon (primers were excluded) is indicated above the alignment. The coloured positions on the sequences indicate nt changes. The arrow indicates a 2 nt deletion present only in CΔ2 alleles. The 3′ ends of cds45 and cds47 are indicated to highlight the absence of cds46. For the maximum-likelihood analysis, values less than 80 % are not shown. All the samples in this figure are classified in the gI group according to the *4a* gene.

The CΔ alleles from five CC samples were cloned, sequenced and compared. Consensus sequences from multiple series of clones from three samples (i.e. 19-115-3, 21-226 and 23-072) were almost identical, indicating a strong epidemiological link despite collection years apart and different sampling sites. DNA patterns were more complex in two other samples from the North region of France (i.e. 23-066 and 23-068). Initially, two distinct phylogenetic groups of alleles, tentatively named CΔ1 and CΔ2, were identified in the pool of clones from these two samples. Within a 406 nt segment, CΔ1 and CΔ2 haplotypes differed by at least 13 SNPs and a 2 nt deletion (identity levels 95.6–96.3 %) (Fig. 5). Notably, CΔ1 sequences closely resembled clones from the other three samples (19-115-3, 21-226 and 23-072). Both CΔ1 and CΔ2 were represented in the two samples 23-066 and 23-068, suggesting a common source of contamination between the two outbreaks.

**Fig. 6.**
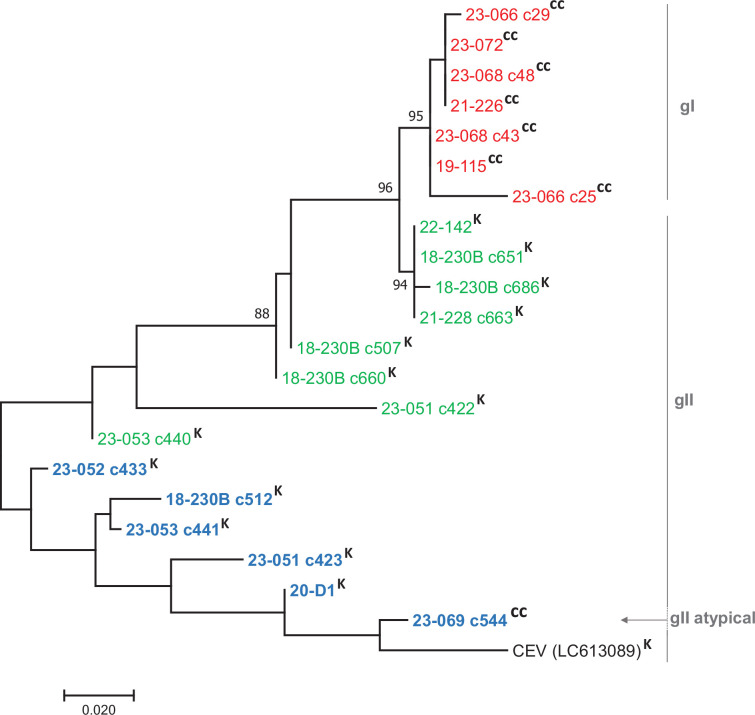
Phylogenetic analysis of a common genetic portion of the cds46 alleles. The first two numbers indicate the year (20 XX) the sample arrived at the ANSES laboratory; the second term refers to the code of the sample and the third term indicates a clone (c) or a consensus of clonal sequences. Sequences of A alleles are shown in green, BΔ alleles in blue and CΔ alleles in red. Only bootstrap values greater than 80% are shown. CC, common carp; K, koi samples. The gI/gII classification (*4a* gene) is indicated at the right of the tree.

To compare the short and complete allele sequences of this study despite their varying sizes, an alignment was created based on a common 233 bp segment situated at the 3′ end of each sequence (Fig. 1). The sequences varied only by SNPs and exhibited similarities ranging from 84.9 to 100%. Virus 23-069 (CC) in particular shared the highest similarity with two viruses from Japanese koi, namely, the reference genome (96.1%) and virus 20-D1 (96.6%). On a phylogenetic tree, two main clusters were observed, albeit with weak bootstrap support (Fig. 6). One cluster comprised sequences of allele BΔ, and the other was composed of alleles A and CΔ. Within this latter cluster, some of the CΔ alleles were closely related to some A alleles, indicating a strong common phylogenetic relationship despite the indel. Furthermore, allele BΔ sequence from sample 18-230B (c512) was clearly distinct (33 SNPs, identity 85.8%) from its homologous portions of allele A from this same sample (4 clones, Fig. 6). Such a large number of substitutions suggest that the deleted allele is not directly derived from the complete allele by a unique deletion event. A similar observation was made for two clones – 1 A and 1 BΔ – obtained from the same sample 23-051, differing markedly in their positions in the tree due to 12 single changes (identity 94.8%).

### Features of the cds46 ORF

The reference sequence of cds46 from Japan exhibited a striking difference compared with our sequences: an extra adenine was present in the reference sequence within a poly-A tract shortly after the putative reference start codon of cds46 (position 46,630). It is unclear whether this nt insertion represents a true variation or an artefact of sequencing commonly found in poly-tracts. Our own sequences, lacking this adenine, suggest an alternative ATG as a start codon, at position 46,544 of the reference genome (LC613089), which is 77 bp upstream of the previously proposed start codon, resulting in a translated product 25 aas longer than the initial one (Fig. 1). The presence of an AT-rich region just upstream (−6 to −68 nt), which harbours various putative promoter motifs, supports the designation of this ATG as a start codon. These putative promoter sequences resemble the consensus AAAAnTGAAAA found in the early promoter region of *Poxviridae*, as well as the motifs TAAA and TAAT described for intermediate and late genes, respectively [22]. A predicted transcription termination signal (TTTTT A/G T), somewhat similar to the T5NT canonical motif used in the vaccinia virus, is located 28 nt downstream of the stop codon.

**Fig. 7.**
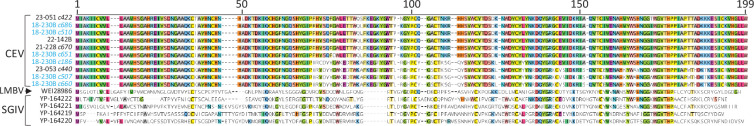
Alignment of the translated products of cds46 A alleles. The sequences shown in blue are from the same sample.

Over the entire length of the 184 aas, the translated products of cds46 of the two groups A1 and A2 exhibit similarities ranging from 78.8 to 100%. The first 32 aas in the NH2 terminus are perfectly conserved, but more variations are observed in the central part of the protein, particularly between aas 63 and 112 (Fig. 7). The function of these longer translation products of alleles A1 and A2 of cds46 is unknown. Searches for homologies with blast revealed some similarities, albeit limited, with several translation products of unknown functions such as ORFs 124R, 125R, 126R and 127R of Singapore grouper iridovirus (SGIV) and LMBV-051 of the largemouth bass virus (LMBV). The sizes of the predicted proteins were similar, and two peptidic motifs, DQYGRxC and GGxWGYTHP, were conserved (Fig. 7).

**Fig. 8.**
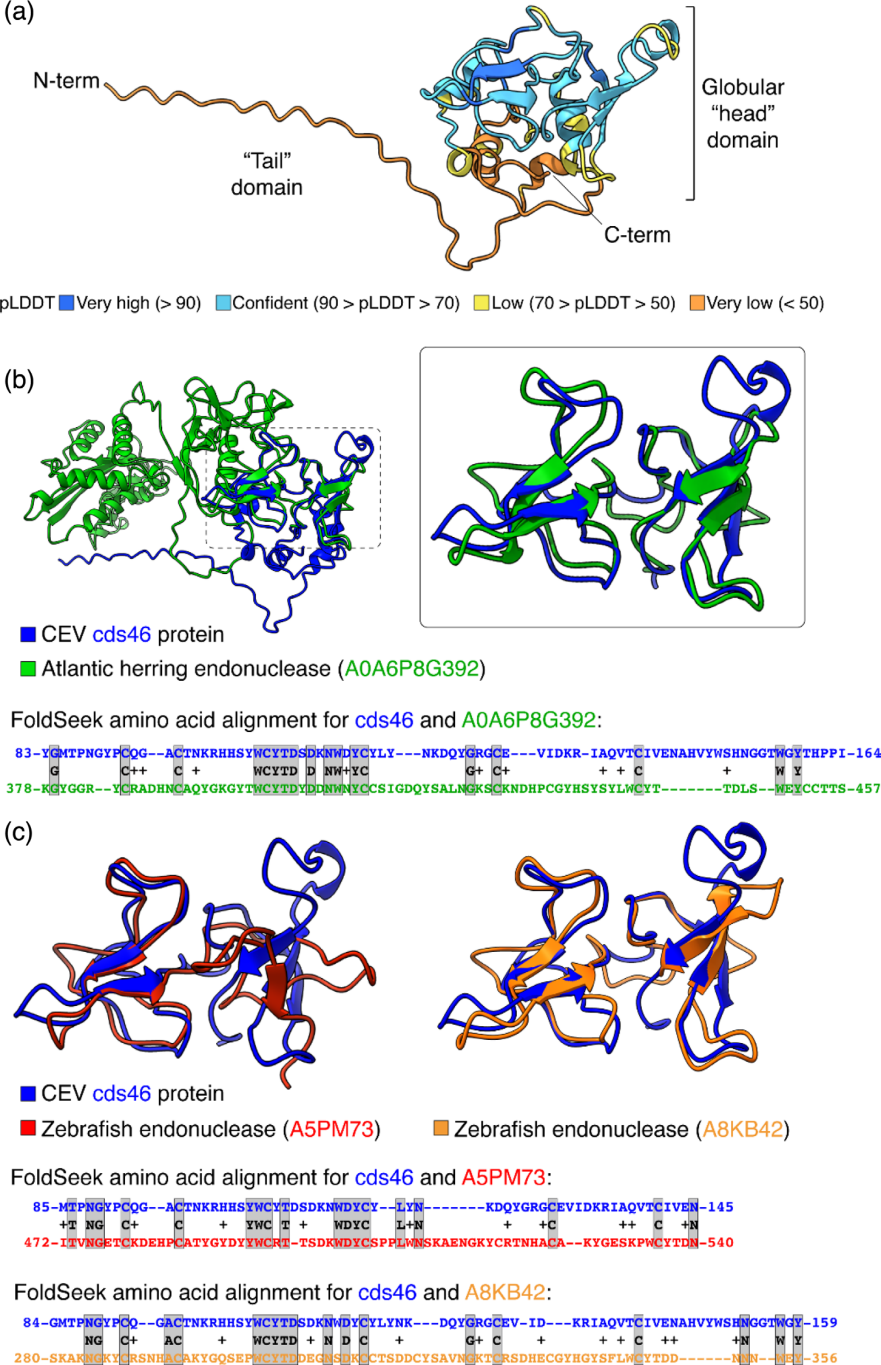
Structural modelling reveals insights into CEV cds46 protein structure–function relationships and origin. (a) AlphaFold prediction of protein structure of CEV cds46. AlphaFold 3 server was used to predict the structure of CEV cds46 protein (A1 allele, sample 23-053). The model is colour coded according to the pLDDT confidence scoring metric. (b) Structural alignment between full-length CEV cds46 protein model and an Atlantic herring (*Clupea harengus*) endonuclease (UniProt A0A6P8G392), the best FoldSeek hit with a GO functional annotation. The region where the proteins align is magnified in the boxed inset. (c) Structural alignments of zebrafish (*D. rerio*) endonucleases (UniProt A5PM73 and A8KB42) with CEV cds46 protein (aa 83-164) identified by FoldSeek. For A0A6P8G392 and A5PM73, the GO functional annotation was found in UniProt, while for A8KB42, the annotation was inferred using protein blast search. (b, c) For each structural alignment, the corresponding pairwise aa sequence alignment is presented below (greyed positions indicate identical residues).

To gain more insights into the protein encoded by cds46, we modelled the structure of one A1 allele (sample 23-053) using AlphaFold (version 3), a machine-learning approach that has revolutionized protein structure prediction [18,19]. Remarkably, AlphaFold modelled a bipartite protein with an unstructured N-terminal ‘tail’ domain (aa 1-31, pLDDT confidence score <50) and a C-terminal globular ‘head’ domain (aa 32-184) composed of several small helices and beta sheets, some of which with very high prediction confidence scores (pLDDT >90) (Fig. 8a). It is worth to point out that the unstructured N-terminal ‘tail’ domain consists mainly of the extra 25 aas that are added to the structured ‘head’ domain due to the presence of the alternative start codon described earlier (Fig. 1). We next used this model as a search query in FoldSeek, a computationally fast and accurate structural search programme capable of rapidly aligning the structure of a query protein against several protein structure databases including the AFDB. Such a structure-based approach can reveal homologies in distantly related proteins even when their primary sequence similarities are low. The top hit with a known function identified by FoldSeek within the AFDB is a cellular protein from Atlantic herring (*Clupea harengus*), with a probability of homology of 1 and *E*-value of 1.14e-2 (UniProt A0A6P8G392). Interestingly, the GO functional annotation indicates that the cellular protein hit is a DNA/RNA non-specific endonuclease. Importantly, while not spanning entire protein lengths (Fig. 8b), the structural alignment of the globular ‘head’ domain of CEV cds46 protein and the cellular endonuclease was striking with a Template Modelling alignment score of ~0.63 (Fig. 8b, box inset). This remarkable structural superposition contrasts with the low primary sequence identity (approximately 26%) found in the aa sequence alignment (Fig. 8b, bottom). Of note, for both CEV cds46 protein and the cellular endonuclease, the structural alignment concerns the C-terminal ends of the respective proteins and does not span the N-terminal endonuclease domain of the cellular enzyme (aa 2-169). To further validate this finding, we narrowed down the FoldSeek search by querying only the highly structured region of cds46 protein (aa 83-164) that was found in the initial structural alignment. This led to the identification of two other high-scoring cellular endonuclease hits (UniProt A5PM73 and A8KB42, probability of homology of 0.89 and 1, respectively) from zebrafish (*D. rerio*), a member of the *Cyprinidae*. Both zebrafish endonucleases and CEV cds46 protein adopted comparable folds with similar structural alignments as found for the Atlantic herring endonuclease, despite primary sequence alignments with low identities (Fig. 8c). We do note some similarities in the cds46 aa alignments with Atlantic herring (A0A6P8G392) and with zebrafish (A8KB42) endonucleases, which share a conserved WCYTD motif with cds46.

### *4a*-based genetic identification

To investigate the genetic links between two distant regions of the viral genome, the 15 CEV-positive samples were also characterized using the partial sequence of the *4a* ORF amplified by PCR. As anticipated, the nine samples from koi clustered within gII, together with other samples from previous studies (Fig. S2). Five samples from CC clustered in gI, each being highly similar to one or another sample identified elsewhere [3]. For instance, sample 23-072 was almost identical to virus 16-061 from 2016. Interestingly, a sixth sample from CC (23-069) was nearly identical to the atypical virus 15-166 described in another study, exhibiting only three substitutions over 890 bp [3]. The high degree of similarity between these latter two samples provides strong evidence of an epidemiological link between the two viruses, which were collected 8 years apart from different regions in France.

Because sample 18-230B showed high genetic heterogeneity when examining the cds46 locus, we decided to further investigate its *4a* locus. This sample is a pool of gills from a batch of 15 koi. Starting from the *4a* amplicon, ten clones were individually sequenced and compared. Three types (a, b and c) of clones were distinguished, with sequences of each type differing from one another by 15 to 41 SNPs (96 –98.5 % identity). Two types, 18-230Ba and 18-230Bb, were each represented by a single clone, and the third type (18-230Bc) was a consensus sequence representing eight clones varying by only one to five SNPs. Notably, type 18-230Ba was nearly identical (one substitution) to another clone (18-230Aa) obtained from a distinct sample of the same batch and previously shown to exhibit some genetic heterogeneity [3]. The two other types recently analysed, 18-230Bb and 18-230Bc, exhibited 8 to 31 substitutions compared to clones 18-230Aa and 18-230Ab analysed elsewhere, confirming a genetic diversity even higher than suspected in this batch of fish. Finally, the finding of a genetic heterogeneity in the *4a* amplicon of sample 18-230B, with at least three groups of variants, was consistent with the three groups identified at the cds46 locus.

To estimate the intra-sample genetic diversity in shipping water, a more detailed analysis of the *4a* fragment was performed on samples of koi imported into France from Japan in 2019 (23-051) and 2020 (20-D1), as well as from Israel in 2023 (23-052 and 23-053). For the Japanese-imported sample 23-051, eight clones were sequenced starting from the amplicon. They differed from one another by zero to six substitutions distributed over the 1074 bp-long amplicon. For one Israeli-imported sample (23-052), sequences from seven clones were nearly identical to one another, with a maximum of five SNPs between two clones. The shipping water from the second Israeli-imported batch of koi (23-053) was more genetically heterogeneous among the 8 clones: 0 to 13 SNPs were observed. Unambiguously, two subpopulations of three and five clones were distinct, each subgroup being different from the other at five positions. Within each subgroup, all the clones differed by a few substitutions dispersed in the rest of the sequences. Sample 20-D1 exhibited some genetic diversity, albeit limited. Of the ten clones, two subpopulations (a and b) of five clones each were visually distinguished. Altogether, the ten clones differed by 1 to eight substitutions. Nevertheless, the consensus sequences of each set of five clones differed by four substitutions, suggesting the coexistence of two variants in this water sample. Although both subpopulations exhibited very high relatedness to the reference Japanese genome, the consensus sequence of 20-D1b differed by only one substitution from the reference Japanese genome (Fig. S2).

To summarize, the analyses of the partial *4a* gene showed that most of the viruses sampled from 2021 to 2023 closely matched viral samples in the database of French carp CEV samples.

## Discussion

Monitoring the geographical spread of CEV strains is crucial for implementing effective control measures and containing the emergence of CEVD in new regions. For other poxviruses such as lumpy skin disease virus, multiple genes and whole genomes have been successfully used to track the spread and genetic evolution of strains [23]. For salmon gillpox virus, a genotyping assay based on simultaneous characterization of eight loci proved valuable for determining the geographic distribution of various lineages and sublineages [24]. Similarly, molecular markers hold promise for studying the genetic diversification and outbreak origin of CEV. Nevertheless, the lack of genomic data has hindered the development of such strategies. In addressing the need for genetic markers, we serendipitously identified an indel associated with the complete cds46 in CEV shed from a batch of koi. Using a newly developed cPCR-based assay, viral genomes without this ORF were identified in samples of different nature and origin. Given the recent publication of the CEV genome, the genetic peculiarity – presented here as a deletion – remains uncertain whether it is really due to a genuine deletion or rather to an insertion. Similar genomic changes between clades of poxviruses have been observed in monkeypox virus, where one clade carries a truncated version of a vaccinia complement control protein, a host range gene, absent in the other major clade [25]. For convenience, we present the cds46 indel as a deletion here, contrasting it with the reference genome. However, it is possible that an ancestral CEV lacking cds46 acquired this gene more recently through horizontal gene transfer, akin to the reported insertion of the entire reticuloendotheliosis virus genome into the fowlpox virus genome [26]. Regardless of the nature of the genomic change – presence or absence of cds46 – both configurations are well represented in our sampling, albeit limited, indicating frequent occurrence or stable dissemination.

Notably, CEV cds46 shows homology with genes found among fish iridoviruses, suggesting a potential origin from a yet-unidentified DNA virus co-infecting *Cyprinus carpio*. Evidence of viral co-infections is common in *Cyprinus carpio*; for instance, sequences resembling *Iridoviridae* and *Orthoherpesviridae* have been detected in the faecal content of apparently healthy CC collected in Mexico and analysed by whole metagenome shotgun, alongside many other viral families. Furthermore, three *Iridoviridae* family member viruses are known to infect carps: LMBV, a *Megalocytivirus* and CC iridovirus [27,29]. Interestingly, cds46 exhibits homology with a gene from LMBV. In addition to *Iridoviridae*, co-infections involving CEV and herpesviruses have been documented in several studies of outbreaks [30,32]. Clearly, the virome of this fish species presents a reservoir of genes potentially implicated in gene transfer with CEV [33].

However, a second hypothesis for the origin of the cds46 gene is suggested by its striking structural similarity to domains of a number of fish proteins, presumably with endonuclease activities. Rather than a direct viral origin, it can be speculated that cds46, and its related genes among several iridoviruses, results from the capture of a host gene by a common viral ancestor, followed by independent evolutions within the *Nucleocytoviricota* phylum. The recruitment (exaptation) of cellular proteins, notably enzymes, for other functions in the viral cycle, is common in a number of viral families, including the *Poxviridae* [34]. For cds46, the major part of the predicted protein shows a strong structural homology only with C-ter domains of several fish proteins, but not to their whole structure. If cds46 really originates from a transfer of a complete host gene inside an ancestral viral genome, a subsequent deletion may have removed a large enzymatic domain before the rest of the sequence diverged between the different viral lineages. Alternatively, only a part of a host gene, corresponding to a structured domain, may have been captured and retained in the viral genome for an as yet-unidentified function. If this scenario is correct, it would represent an extension to the depth of protein domains for the exaptation concept [35].

While the species that are revealed using such an approach do not necessarily reflect the actual source of the exapted protein [34], these results indicate that the cellular endonucleases identified are conserved in different fish species, including zebrafish, a member of the *Cyprinidae* like *Cyprinus carpio*. In addition, we have confirmed that both zebrafish endonucleases have orthologues in CC by searching through the Genomicus comparative genomic database [36]. In summary, the above analyses raise the possibility of an exaptation event that occurred in an ancestral virus and involved a cellular gene, encoding a structurally conserved endonuclease, which then continued to evolve independently in a distinct evolutionary path within the viral genome.

On the basis of our structural modelling and FoldSeek analyses suggesting that the C-terminal globular ‘head’ domain of cds46 is structurally related to the C-terminal domain of cellular endonucleases of fish species, we reasoned that cds46 could be involved in binding to DNA. Further modelling of cds46 using AlphaFold 3, which can predict joint structures of complexes including proteins, DNA, RNA and other molecules, suggests that it could interact with dsDNA (not shown). Interestingly, only the dimeric form of cds46 was predicted to interact with DNA but not its monomeric form. While these predictions await experimental validation, they highlight the power of the predictive structural bioinformatics, which can guide the formulation of hypotheses that can then be tested experimentally in order to address the function of newly characterized proteins, such as cds46.

The prediction that cds46 can form dimers that bind to DNA could be tested in the future by classical biochemical assays, such as electrophoretic mobility shift assay, provided that the cds46 protein can be expressed heterologously and purified. To further shed light on its function, it would also be useful to transiently express cds46 in a carp cell line maintained *in vitro* and subsequently determine its localization within cellular compartments. Alternatively, expressing cds46 in a poxvirus system under its own promoter, but in a non-fish cell line – since no fish poxvirus can be cultured *in vitro* to date – could provide insights into its natural expression timeline and localization. However, these recombinant models may not fully replicate potential interactions with the hundreds of predicted CEV proteins. Therefore, the *in situ* localization of the expression of cds46 mRNA (or protein) could be performed with specific probes (or antibodies) on koi tissues conserved during an outbreak of CEV. Finally, elucidating the function of the SGIV cds46 homologue, which can be cultured *in vitro* and genetically modified, could offer complementary insights into the function of CEV cds46 [37].

Along with SNPs and traces of recombination events, another type of deletion was observed in several new viral sequences, indicating important genetic variation in this region. We characterized multiple cds46 alleles that varied in the size of the deleted region and in the presence of SNPs. The main haplotypes, A1 and A2, possess the complete cds46 ORF but differ in their SNP profiles. Among the other alleles, BΔ alleles exhibited a substantial deletion and CΔ alleles were defined by a large deletion, although shorter than those of BΔ, and included a sequence segment with no homology to any other sequence.

This is the first report of the existence of CEV variants with large deletions. In a previous study, a small triplet deletion was found in the *4a* ORF, which was associated with a microsatellite, but did not affect the presence of the ORF. In the case of cds46, the entire ORF may be absent, regardless of the considered start codon – either the one identified in the reference sequence or one upstream, which appears more likely in our sequences. Gene loss has been documented in several members of the *Poxviridae* family [38]. For *Orthopoxvirus* members, gene loss is a common mutation type that influences the virus–host relationship and evolution, potentially restricting the host range of a virus or confining the mutated virus to particular environmental niches [39,40]. For example, gene loss observed in variola virus may be linked to a restricted human host range. If CEV undergoes such evolution, *Cyprinus carpio* may be the only highly susceptible host due to gene reduction from ancestral viruses that had a wider host range. However, because the exact function of the CEV cds46 gene remains unknown, predicting the effect of its absence on virus biology is currently impossible. Nevertheless, regular monitoring for the presence of this gene will be crucial in the future to assess changes in the frequency of the alleles showing this deletion. CEV emerged in many different regions and environments decades ago, and its genome is expected to mutate and adapt to new carp populations and changing chemical and physical conditions. More complete CEV genomes, particularly those of gI, will be essential for detecting evidence for this potential gene reduction.

The two types of deletions that gave rise to the BΔ and CΔ alleles are likely the results of independent events, because (i) the sizes of the deletions are different; (ii) a fragment of allele CΔ lacks a homologous sequence, suggesting an additional insertion event – possibly through non-homologous recombination and (iii) the shared sequences differ by a significant number of SNPs. Nonetheless, the possibility of the occurrence of an initial large deletion from an ancestral strain cannot be excluded, potentially creating a deleted haplotype that subsequently evolved into different variants, including one with an insertion of a non-viral sequence. Alleles A and BΔ are predominantly found in koi and are associated with gII, based on *4a* sequences. In contrast, CΔ alleles were identified only in CC and associated with *4a*-based gI. Further characterization of additional CC samples is necessary to confirm this association. If CΔ alleles indeed show a strong genetic association with gI, the proposed new cPCR could serve as a useful and straightforward test to identify the *4a*-based genogroup of any positive sample without requiring sequencing.

Interestingly, we observed the co-occurrence of two amplicons of different sizes, A and BΔ, in four samples. For these particular samples, it was not possible to determine whether this pattern reflected a mixed infection in individual fish or single infections of distinct fish transported together in the same bags, or both. Two of these samples consisted of shipping water from bags containing batches of tens to hundreds of koi fish. Therefore, it is likely that the water contained viral DNA (free or in particles) produced by several animals infected with one more variant. The third sample, 18-230B (pooled koi gills), was particularly complex, containing three alleles – A1, A2 and BΔ – according to cPCR results and sequencing of several A amplicon clones. In a previous study, a sample from the same batch (18-230A) was already suspected to carry genetic heterogeneity with distinct variants of the *4a* fragment. Given that 18-230B was obtained from a pool of tissues from farmed koi, the diversity of sequences can be explained by co-infection of individuals, or single infections of different fish, or both. This scenario was similar for the fourth sample (21-228), which consisted of a pool of organs from eight koi and two amplicons. Intra-sample molecular diversity was also demonstrated in two individual CCs from two different outbreaks in northern France (i.e. 23-066 and 23-068). Despite the absence of any hydrological connection between the two ponds, these two fish carried the same two alleles CΔ1 and CΔ2, which differed by a number of substitutions. A likely explanation is the introduction of already co-infected fish from the same origin into both ponds. Another hypothesis is the transfer of infected fish from the first pond to the second one, by birds or anglers. Whatever the link between the two ponds, these cases provide the first evidence of possible co-infection in the same animal by two types of CEV gI.

Therefore, all instances of mixed infections underscore the importance of testing pools of fish or volumes of shipping water to comprehensively explore the diversity of haplotypes present within a batch moved from one site to another. This is particularly important for molecular tracing when comparing viral populations between an imported batch and fish randomly sampled after an outbreak. Without a thorough assessment of the viral populations before and after outbreaks, false conclusions can be drawn if different molecular markers are found between the CEVs found in newly introduced carps and those affected by the disease. Both CC and koi often undergo intensive circulation and batch mixing due to commercial activities. These practices justify the implementation of screening strategies to trace the dissemination of haplotypes. Moreover, apart from the interest in elucidating the origin of outbreaks, it would also be valuable to investigate whether deleted and non-deleted alleles of cds46 can co-infect a single fish. Such co-infections are necessary for recombination events to occur.

The cds46 region of virus 23-069 (CC) differed from three other CC samples, despite all being associated with outbreaks in the same region of France in spring 2023. Unlike the three other CC viruses classified as CΔ, the cds46 allele of 23-069 belonged to the BΔ group, similar to alleles found in koi fish. This difference further coincides with the fact that all four outbreaks were linked to restocking ponds with CC or other fish species purchased from different suppliers. Interestingly, based on the partial *4a* fragment, sample 23-069 showed further differentiation from the viruses found in CC in the present study, being more closely related to, although still distinct from gII. It also showed a resemblance to the atypical sequence CEV 15-166, identified once in France in 2015, but which has never re-emerged since. This indicates genetic relatedness of CEV 23-069 to gII across two distant regions of the genome, despite several SNPs and infection of CC. Unfortunately, no additional DNA was available from virus 15-066, preventing a comparison of its cds46 sequence with that of 23-069. Nevertheless, our results clearly demonstrate that cds46 can effectively distinguish gII and gII-like sequences from gI sequences. Therefore, this marker will be useful for screening atypical haplotypes in CC and to monitor their prevalence nationwide.

The comparison of different cds46 sequences revealed hybrid molecules, possibly resulting from recombination events between haplotypes. Such genetic changes are common in poxviruses and have already been implicated in the atypical pattern of CEV 15-066 in the *4a* gene [3,38]. If recombination indeed underlies the atypical pattern of CEV 15-066, the detection of a very similar genotype 8 years later (virus 23-069) demonstrates that this type of genetic evolution produces viable viruses capable of dissemination and associated with CC deaths. Our observations also suggest that CEV gII from koi are likely subject to genomic exchanges in the cds46 region, which seems to be a hotspot for various mutations: substitutions, indels and recombination. Regarding recombination, the presence of several haplotypes in the same batch of fish or in individual fish shows that the conditions necessary for recombination are met – namely, the replication of homologous molecules in the same cells.

In conclusion, we demonstrated here that a specific genomic region, cds46, is valuable as a molecular marker for differentiating samples and haplotypes of CEV. More broadly, the ancestral link between cds46 ORF and cellular endonucleases revealed by predictive structural analyses provides new insights into poxvirus genomic plasticity and evolution. Complete sequencing of more CEV genomes, particularly those belonging to gI, will likely uncover other genomic regions with similar potential.

## supplementary material

10.1099/jgv.0.002048 Uncited Supplementary Material 1.

## Abbreviations

aa: amino acid
AFDB: alphafold database
BLAST: Basic Local Alignment Search Tool
CC: common carp
CEV: carp edema virus
CEVD: carp edema virus disease
cPCR: conventional PCR
GO: gene ontology
KSD: koi sleepy disease
LMBV: largemouth bass virus
nt: nucleotide
ONT: Oxford Nanopore Technologies
ORF: open reading frame
PCR: polymerase chain reaction
