# Supplementary material for: cds46, a highly variable carp edema virus gene
Source: J Gen Virol. 2024 Nov 20;105(11):002048. doi: 10.1099/jgv.0.002048 (PMC11578112; doi:10.1099/jgv.0.002048)
Supplement: Uncited Supplementary Material 1. [file jgv-105-02048-s001.pdf]

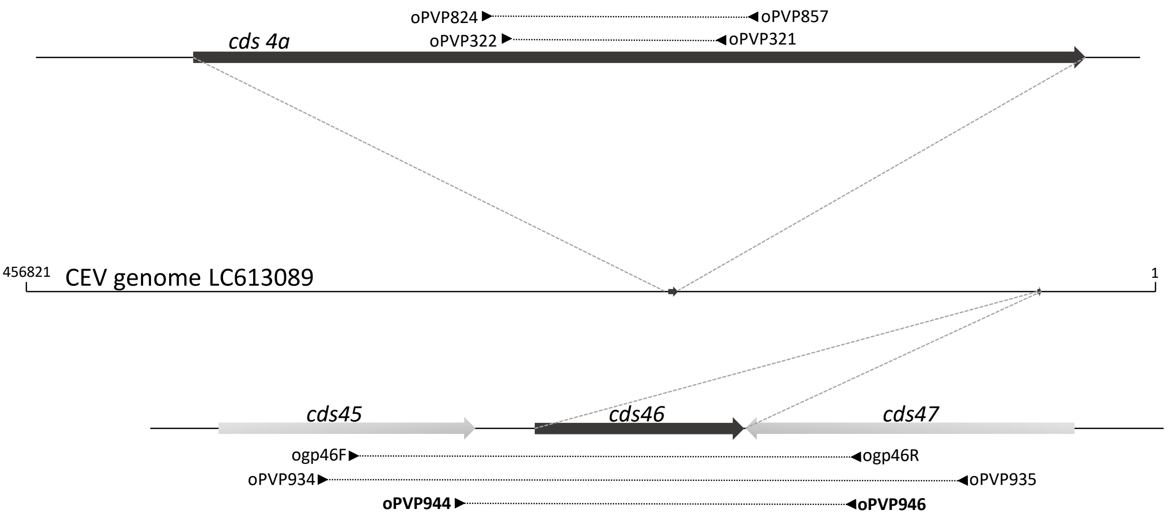

Primer name	Sequence (5'-3')	Amplicon size (expected for CEV LC613089)	Gene targeted
oPVP321*	AGTCCAAGAGTTTTCTTCTC	870 bp	4a
oPVP322*	TGGACAAGGTAATTATGAGC		
oPVP824	GTGGTAACTTTACTTGTCCTCC	1074 bp	4a
oPVP857	GTACTTTATTTGCTGCAGGAT		
oPVP934	TCTGTGCAAAATCTGACTGC	1481 bp	cds46
oPVP935	CGAATGTGATCCACTTATTCC		
oPVP944	GTCAATTATTTGGAATTTCTAATCC	920 bp	cds46
oPVP946	CCTCCTTCTCCRATTGATCTTAG		
ogp46f	CTACCATGACTCTGTTTGCTAC	1175 bp	cds46
ogp46r	TTGAATCCTCCTCCTTCTCC		

Supplementary figure S1. **Sequences and positions of oligonucleotides on the genetic map of CEV**. (*) refers to primers not used in the present study and mentioned just for comparison with the new sets (Baud *et al*. 2021). In order to present the cds in the 5'-3' orientation, the genome is depicted as the reverse complement of its Genbank reference.

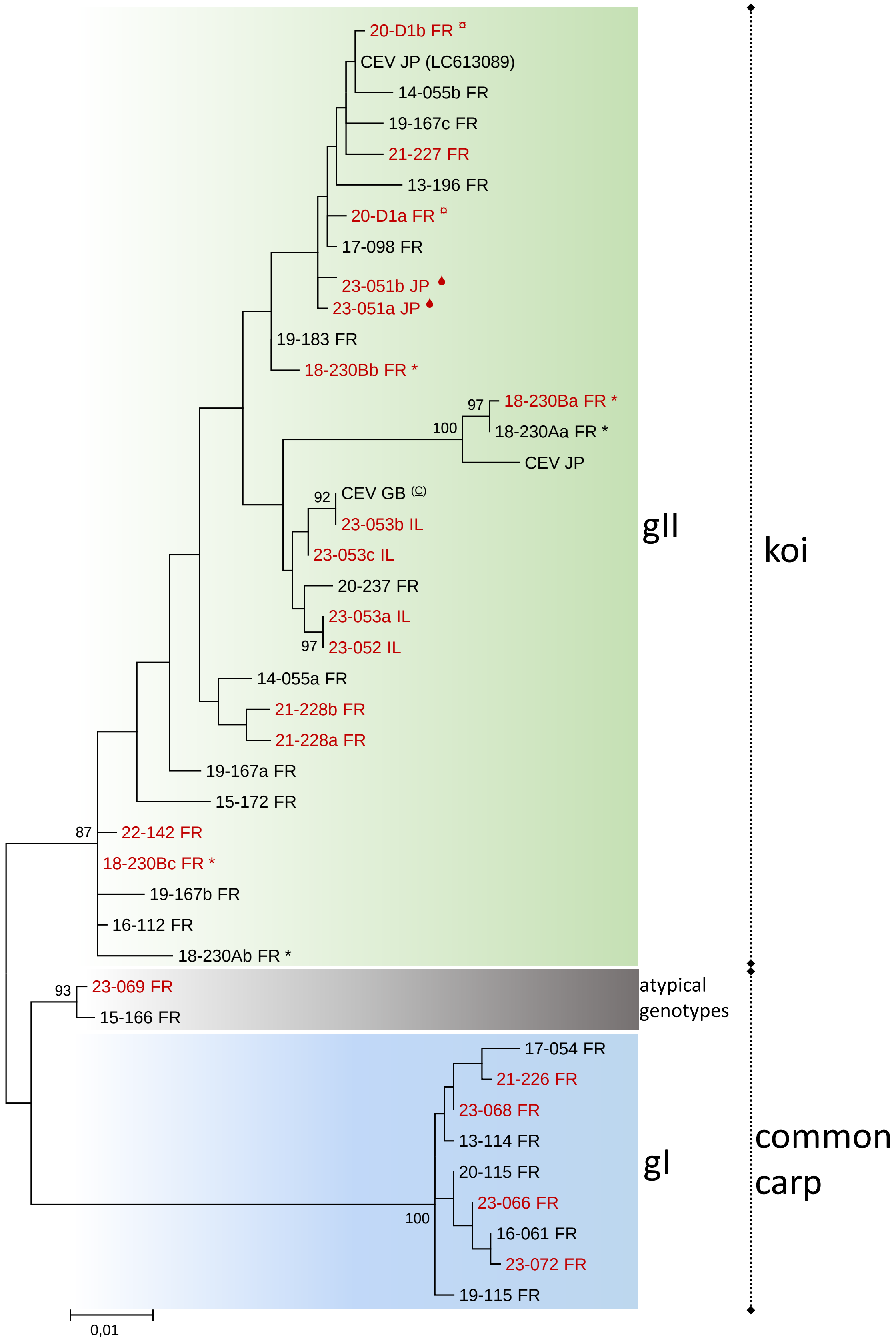

Suppl. fig. S2. Phylogeny of partial 4a sequences. The two first numbers indicate the year sample arrived at the ANSES laboratory, followed by the reference of the sample and the country of origin. The sequences were obtained directly from amplicons or consensuses of cloned amplicons exhibiting variations (a, b, etc.). Sequences obtained in the present study are shown in red. The sequences 18-230Aa,b correspond to 18-230a,b previously published (Baud *et al.* 2021). The underlined C is for a sequence from UK exhibiting a cytosine at a particular position (Baud *et a*l 2021). The symbols *, ¤ and ♦ indicate sequences from samples of a same batch of fish.

Sample	GenBank partial 4a	GenBank locus cds46
18-230B	PP971877<br>PP971878<br>PP971879	PQ007470<br>PQ007471<br>PQ007472<br>PQ007473<br>PQ007478<br>PQ007479<br>PQ007481<br>PQ007488
19-115-3	Baud *et al.*, 2021	PQ007502
20-237	Baud *et al.*, 2021	ns
21-226	PP971882	PQ007497
21-227	PP971883	ns
21-228	PP971884<br>PP971885	PQ007475<br>PQ007476
22-142B	PP971886	PQ007474
23-051	PP971895<br>PP971896	PQ007477<br>PQ007489
23-052	PP971893	PQ007482<br>PQ007483<br>PQ007484
23-053	PP971891<br>PP971892<br>PP971894	PQ007480<br>PQ007485<br>PQ007486<br>PQ007487
23-066	PP971887	PQ007492<br>PQ007493<br>PQ007494<br>PQ007499<br>PQ007500<br>PQ007501<br>PQ007503<br>PQ007504<br>PQ007510
23-068	PP971888	PQ007495<br>PQ007498<br>PQ007505<br>PQ007506<br>PQ007507<br>PQ007508<br>PQ007509
23-069	PP971889	PQ007490
23-072	PP971890	PQ007496
20-D1	PP971880<br>PP971881	PQ007491

Supplementary table. Genbank references of the sequences produced in the present works. ns, amplicons not sequenced
